# Characterization of the complete mitochondrial genome of the snow crane-fly *Chionea crassipes gracilistyla* (Diptera, Tipuloidea, Limoniidae) with phylogenetic analysis

**DOI:** 10.1080/23802359.2019.1643796

**Published:** 2019-07-22

**Authors:** Zehui Kang, Xiao Zhang, Ding Yang

**Affiliations:** a Key Lab of Integrated Crop Pest Management of Shandong Province, College of Plant Health and Medicine, Qingdao Agricultural University, Qingdao, China;; b Department of Entomology, China Agricultural University, Beijing, China

**Keywords:** Mitochondrial genome, phylogeny, *Chionea*, snow crane-fly

## Abstract

The genus *Chionea* Dalman, 1816 is a peculiar group of crane flies with extremely short wings. In this study, we report the first complete mitochondrial (mt) genome sequence of the genus *Chionea*, which is a circular molecule of 15,775 bp with an AT content of 76.9% and contains 13 protein-coding genes, 22 tRNA genes, 2 rRNA genes, and a long non-coding region. Phylogenetic analysis revealed that the Pediciidae was sister-group to the remaining Tipuloidea and strongly supported the sister-group relationship between Cylindrotomidae and Tipulidae.

The genus *Chionea* Dalman, 1816 is a peculiar group of crane flies known as the snow crane flies. Its general characters are given by Byers (1983) and Zhang et al. (2012). Members of the genus often move on snow-covered surfaces during warm days in winter. Sometimes they may be found under moss and stones, among fallen leaves or in the nests of small mammals (Schmitz 1914). Forty *Chionea* species dispersed over the northern hemisphere, of which 22 species are from the Palaearctic Region and 18 species are from the Nearctic Region (Oosterbroek 2019).

The superfamily Tipuloidea is one of the most taxonomically diverse groups of flies with more than 15,000 known species. Starý (1992) divided it into four families: Pediciidae, Limoniidae, Cylindrotomidae, and Tipulidae. However, Petersen et al. (2010) considered that the existing four major higher-level groups of Tipuloidea were not natural monophyletic groups. The phylogeny and classification of Tipuloidea are controversial and unresolved for a long time and the mitochondrial (mt) genomes are useful molecular techniques for systematic studies. Here, we report the first complete mt genome sequence of the genus *Chionea*, which will provide an insight into the phylogeny of Tipuloidea.

The specimen of *C. crassipes gracilistyla* Alexander, 1936 was collected from Mt. Tianhua in Liaoning province of China (41°4′N, 124°35′E) and stored in the Entomological Museum of China Agricultural University (No. DTip-002). The total DNA was extracted from the thoracic muscle of individual specimen using the TIANamp Genomic DNA Kit (TIANGEN, Beijing, China). The mt DNA fragments were amplified using standard primers for insects and another eight primers were designed to amplify the nonconservative sequences. The sequence was annotated manually following the method proposed by Cameron (2014). BI analyses were conducted using PhyloBayes under the heterogeneous model CAT-GTR (Lartillot et al. 2013).

The whole mt genome of *C. crassipes gracilistyla* (GenBank accession no. MK941181) is 15,775 bp long with an AT content of 76.9%. It contains a typical gene content: 13 protein-coding genes, 22 tRNA genes, 2 rRNA genes, and a long non-coding region. The gene order shows a conserved arrangement pattern with 23 genes transcribed on the majority strand and 14 genes encoded on the minority strand. Three conserved overlapping regions are found in this mitogenome: 8bp between *tRNA^Trp^* and *tRNA^Cys^*, 7bp between *ATP8* and *ATP6*, and 7bp between *ND4* and *ND4L*. Furthermore, one non-coding conserved intergenic region which often existed in dipteran insects is found. It is 12 bp long and located between *tRNA^Ser(UCN)^* and *ND1*. The longest non-coding intergenic region in the mt genome is 1048 bp in length.

The phylogenetic tree in our study (Figure 1) indicated that the Pediciidae was sister-group to the remaining Tipuloidea, which was accepted by Ribeiro (2008), Petersen et al. (2010) and Kang et al. (2017). Our phylogenetic analysis also showed a strong support for the sister-group relationship between Cylindrotomidae and Tipulidae. In addition, Limoniidae is not supported as a monophyletic clade and this result is concordant with Petersen et al. (2010) and Zhang et al. (2016).

**Figure 1.**
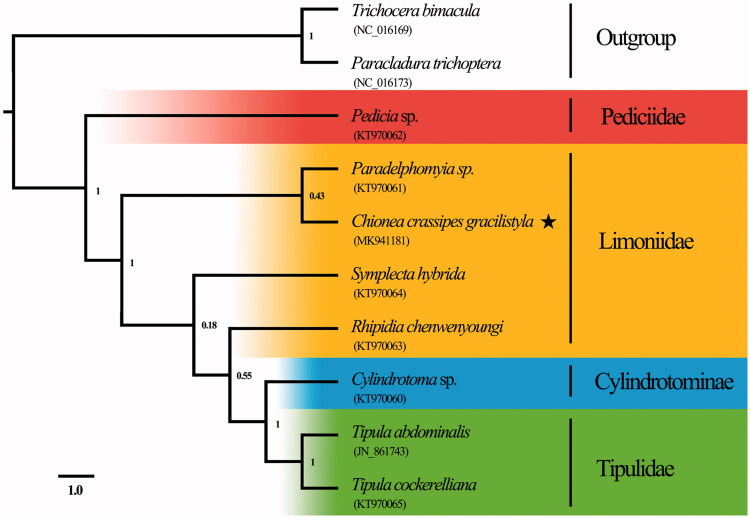
Phylogenetic tree of Tipuloidea based on whole mitochondrial genomes using PhyloBayes under the heterogeneous model. Numbers above the branches are posterior probabilities. GeneBank accession numbers of each species were listed in the tree.
